# Expression of matrix metalloproteinase-12 in aortic dissection

**DOI:** 10.1186/1471-2261-13-34

**Published:** 2013-05-03

**Authors:** Yi Song, Yuehui Xie, Feng Liu, Chong Zhao, Rui Yu, Shao Ban, Qiufang Ye, Jianxion Wen, Haibo Wan, Xiang Li, Runwei Ma, Zhaohui Meng

**Affiliations:** 1 Laboratory of Molecular Cardiology, Department of Cardiology; The First Affiliated Hospital Of Kunming Medical University, Kunming, 650032, China; 2 The Department of Cardiovascular Surgery, The Second Hospital of Yunnan Province, Kunming, 650032, China; 3 Department of Computer Science, The Faculty of Basic Medicine, Kunming Medical University, Kunming, 650031, China

**Keywords:** Aortic dissection, MMP-12, Protein expression

## Abstract

**Background:**

Aortic dissection(AD) is an acute process of large blood vessels characterized by dangerous pathogenic conditions and high disability and high mortality. The pathogenesis of AD remains debated. Matrix metalloproteinase-12 (MMP-12) participates in many pathological processes such as abdominal aortic aneurysm, atherosclerosis, emphysema and cancer. However, this elastase has rarely been assessed in the presence of AD. The aim of the present study was to investigate the expression of MMP-12 in aortic tissue so as to offer a better understanding of the possible mechanisms of AD.

**Methods:**

The protein expression levels of MMP-12 were analyzed and compared in aorta tissue and the blood serum samples by reverse transcription polymerase chain reaction(RT-PCR), Western blotting, immuno-histochemistry, fluorescence resonance energy transfer ( FRET ) activity assay and enzyme-linked immuno sorbent assay ( ELISA ), respectively. Ascending aorta tissue specimens were obtained from 12 patients with an acute Stanford A-dissection at the time of aortic replacement, and from 4 patients with coronary artery disease (CAD) undergoing coronary artery bypass surgery. Meanwhile, serum samples were harvested from 15 patients with an acute Stanford A-dissection and 10 healthy individuals who served as the control group.

**Results:**

MMP-12 activity could be detected in both AD and CAD groups, but the level in the AD group was higher than those in the CAD group (P < 0.05). MMP-12 proteolysis existed in both serum samples of the AD and healthy groups, and the activity level in the AD group was clearly higher than in the healthy group (P < 0.05). For AD patients, MMP-12 activity in serum was higher than in the aorta wall (P < 0.05). MMP-12 activity in the aortic wall tissue can be inhibited by MMP inhibitor v (P < 0.05).

**Conclusion:**

The present study directly demonstrates that MMP-12 proteolytic activity exists within the aorta specimens and blood samples from aortic dissection patients. MMP-12 might be of potential relevance as a clinically diagnostic tool and therapeutic target in vascular injury and repair.

## Background

Acute aortic dissection(AD) is the most common aortic catastrophe. The overall in-hospital mortality rate for acute aortic dissection in China is 17.7%, with most of the deaths occurring during the first week [1]. In the United States, aortic dissection and aneurysms rank among the 15 leading causes of death for all Americans [2], and are responsible for nearly 14,000 deaths each year [3]. Patients with acute type A dissection require emergency surgery, as the death rate in acute dissection may be as high as 1% per hour during the first 24 hours [4]. The basic pathological change of AD is degeneration of the aortic media. Matrix metalloproteinases (MMPs) are a major family of enzymes that degrade various components of the extracellular matrix and basement membrane of the aortic wall, but the key effectors and mechanism of its action remain unknown [5]. A large number of metalloproteinases have been identified in degenerative aortic aneurysms [6], but relatively few studies have focused on the role of MMPs in AD. Several investigators, applying both immunohistochemical techniques and zymography on acute dissection specimens, have reported increased MMP-9 and −2 expression at the site of the initial tear in patients with aortic dissection [7,8]. A recent study by Akiyama and colleagues showed that MMP-2 and MT1-MMP might be involved not only in the degeneration of aortic dissection but also in tissue remodeling [9]. Tissue inhibitors of matrix metalloproteinases (TIMPs) are the dominant inhibitors of MMPs and are able to control MMP-mediated ECM breakdown by binding active or latent forms of MMPs in a 1:1 molecular ratio [10]. The balance between MMPs and TIMPs are critical for the eventual ECM remodelling in human aortic tissue [11,12]. In contrast, the former researchers [13] showed that the expression of MMP-2 and TIMP-2 in aortic samples from patients with acute aortic dissection were significantly lower than in aortic samples from patients undergoing coronary artery bypass, implied that an imbalance between TIMP-2/MMP-2 and TIMP-2/MMP-9 might play an important role in the onset of aortic dissection.

Considering that MMPs play a central role in matrix degradation and aneurysm progression, it seems that these proteinases constitute attractive targets of drug treatment. Over the past few decades , selective inhibitors of MMPs have been developed, but in most cases these compounds have not met the promise and expectations in clinical trials [14-16]. A multicentre clinical study [17] found that a long-term treatment with doxycycline, a synthetic tetracycline derivative known to inhibit MMPs nonselectively, was associated with reduced plasma MMP-9 levels in patients with small asymptomatic abdominal aortic aneurysms. Therefore, administration of doxycycline is expected to be an attractive option for aneurysmal disease pharmacotherapy.

MMP-12 (matrix metalloproteinase12), also known as macrophage metalloelastase, was first identified as an elastolytic metalloproteinase secreted by inflammatory macrophages 30 years ago [18]. MMP-12 is synthesized as an inactive zymogens (54 kDa), and is quickly activated through autolytic cleavage in the propeptide domain to yield a proteolytically avtive 45 kDa form, which then breaks down to the common 22 kDa form [19]. Although the major substrate for MMP-12 is elastin [20], which is abundant in the lung and arterial wall, MMP-12 is able to degrade a broad spectrum of ECM components such as collagen type IV, fibronectin, laminin, vitronectin, proteoglycans, and plasminogen [20]. Increased activity of MMP-12 from inflammatory macrophages is associated with abdominal aortic aneurysm [21], atherosclerosis [22], and emphysema [23].

Although there are substantial numbers of reports on the localization of several MMPs in aortic aneurysms, little is known about the roles of MMPs in the pathogenesis of AD. In this study, using immunohistochemical and biochemical methods, the expression of MMP-12 and its roles in AD was examined in the aorta and blood serum samples of patients with AD. The results present new evidence that MMP-12 is involved in the development of AD.

## Methods

The institutional research ethics committee of Kunming Medical University approved the study, and written informed consent was obtained from each subject. This study was conducted in accordance with the ethical requirements.

### Materials

Intraoperative aortic samples were obtained from 12 patients with Stanford Type A acute aortic dissection as experimental group. These patients received Bentall operation in the period from April 2010 to June 2011 at the No. 1 Hospital Affiliated to Kunming Medical University. 9 were male and 3 were female (mean age, 44.7 years); 8 patients (66.67%) had a history of hypertension and 7 patients (58.33%) used to smoke. All of them had a color Doppler ultrasonic examination and computed tomography and all were diagnosed as acute aortic dissection Stanford A. Control aortic tissues were obtained from 4 cases of CAD without aorta wall damage who underwent coronary artery bypass surgery; samples were taken by punching a hole on the aortic wall tissue with a hole punch. All patients with CAD were male, aged 65.5±3.3; 2 patients had a history of hypertension and 3 patients had a history of smoking. Both groups were ruled out of the possibility of inflammation of the chest cavity, Marfan syndrome, bicuspid aortic valve abnormalities, cardiomyopathy, chronic pulmonary heart disease and infectious endocarditis possibilities.

Serum samples were obtained from 15 patients with Stanford type A acute aortic dissection as the experimental group. All patients were hospitalized in the period from April 2010 to June 2011 at No. 1 Hospital Affiliated to Kunming Medical University. Ten age-matched healthy people were included as a control group.

**Figure 1:**
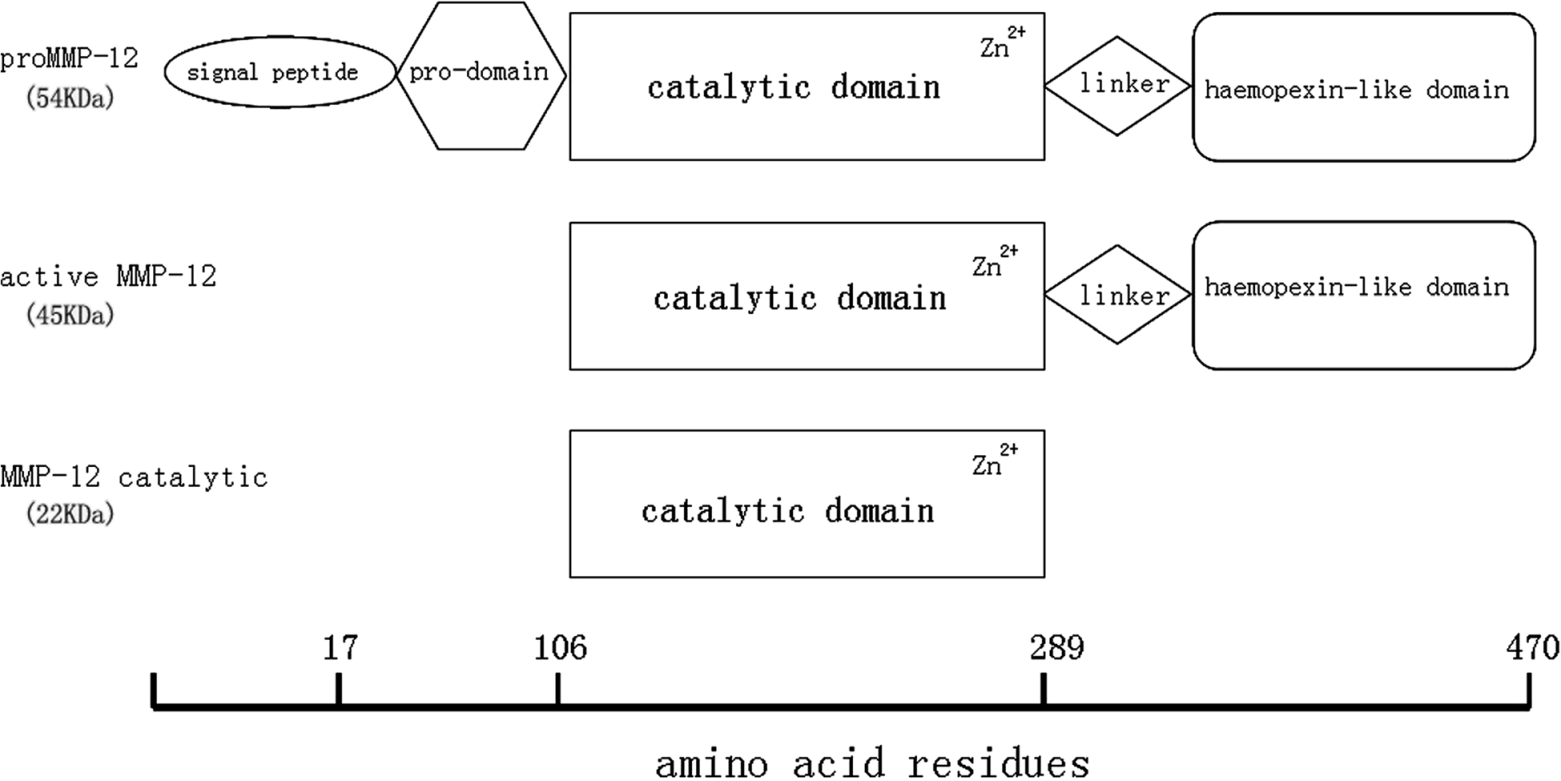
**The domain structures of human MMP-12.** The latent form of human MMP-12(top), the active form of MMP-12 with molecular weight 45 KDa (middle), and the catalytic domain of MMP-12 with molecular weight 22 KDa (bottom) are illustrated. The precise amino acid residues are known from protein sequencing.

Recombinant human MMP-12 (GenBank: AAI43774.1) was obtained in our laboratory. Briefly, the prokaryotic plasmid pET -17×b - MMP-12 was constructed and transformed into E. coli strain BL21 (DE3). The recombinant protein expressed in *E. coli* consisted of 455 amino acid residues from Leu17 to Cys470 (Figure 1), including the prodomain, catalytic domain, the junction between catalytic domain and hemopexin domain, and the hemopexin-like domain. Recombinant human MMP12 (54 KDa ) was expressed in the form of inclusion bodies. After refolding, it underwent self-activation to generate two active forms with molecular weights of 45 KDa and 22 KDa.

### Reverse transcription-polymerase chain reaction

Aortic wall tissues (400 mg) was ground to a fine powder using a mortar and pestle in liquid nitrogen, to which a certain amount of Trizol (Gibco Brl, Rockville MD, USA) was added to extract RNA according to the manufacturer’s instructions. Approximately 1.5 μg of total RNA from each sample was used to conduct reverse transcription reaction in a 50 μl volume using the RNA PCR KitVer.3.0 (Takara Biotechnology, Dalian, China). The reaction mixture was incubated at 42°C for 2 h and the reaction was terminated by heating to 99°C for 5 min. The synthesized cDNA was used for PCR amplification or stored at −80°C for further analysis.

PCR primers (GenScript, Nanjing, China) were designed to amplify MMP-12 cDNA. The forward primer 5'-CGATGAGGACGAATTCTGGACTAC-3' is situated in the exon 4, and the reverse primer 5'-GGTTCTGAATTGTCAGGATTTGGC-3' is situated in the exon 6. The primer sequences correspond to residues Asp211 to Pro292 in the catalytic domain of human MMP-12. The PCR reaction was performed in a 50 μl volume containing 0.5 mM of each primer, 5 μl PCR buffer, 0.5U Ex-Taq DNA polymerase (Takara Biotechnology, Dalian, China). Reaction conditions included initial denaturation at 94°C for 2 min, followed by 35 cycles at 94°C for 1 min, annealing at 55°C for 1 min and extension at 72°C for 1 min, followed by a 10 min final extension at 72°C. PCR products were separated on 1.0% agarose gels and visualized by Gelview (Bioteke,Beijing,China) staining.

The quality of the total RNA was determined by RT-PCR for the house-keeping gene GAPDH (glyceraldehyde-3-phosphate dehydrogenase). The primer sequences were as follows: GAPDH sense, 5'-CCCATCACCATCTTCCAGGAGCG-3'; anti-sense, 5'-GGCAGGGATGATGTTCTGGAGAGCC-3' (GenScript, Nanjing, China). The PCR reaction contained 10.0 μl of cDNA, 0.5 μM of each primer, 5 μl PCR buffer, 0.5 U Ex-Taq DNA polymerase in a final volume of 50 μl using the following conditions: 95°C for 5 min, 34 cycles of 94°C for 30 s, 55°C for 30 s, and 72°C for 1 min, with a 10 min final extension at 72°C.

### Immunohistochemical staining

Aorta wall tissues (4 cm)were obtained at the aortic dissection site, The blood was washed from the newly obtained sample with 0.9% sodium chloride, dipped the sample into 10% Formalin solution for 24 h for serial sections of 4 μm thick, then, stained with hematoxylin for observation. The antibody for MMP-12 (Epitomics, Inc, CA, USA) was a rabbit monoclonal antibody generated by immunizing rabbit with the synthetic peptide corresponding to residues on the C-terminus of human MMP-12; After incubating with Biotin-conjugated goat anti-rabbit IgG (Santa Cruz Biotechnology, Santa Cruz, CA, USA) for 15 min at room temperature(RT), the streptavidin-alkaline phosphatase-biotin complex solution was applied to all sections for 15 min according to the manufacturer’s instructions. Positive results of immunohistochemistry indicated yellowish brown or brown particles presented in the cytoplasm. A random selection 10 fields were viewed under a 400× microscope and calculated the percentage of positive cells was calculated. Rating criteria: no positive expression scored 0 point (negative), positive expression less than 25% scored 1 point (weak positive), positive expression higher than 25% and lower than 50% scored 2 points (positive) and positive expression higher than 50% scored 3 points (strong positive).

### Western blotting

Aorta wall tissue (400 mg) was obtained and ground into powder in liquid nitrogen, to which 4 ml Trizol was added and mixed, then it was centrifuged at 11,000 rpm for 15 min. The supernatant was then spun for an additional 10 min and collected. The protein (15 μl) from the tissue samples was immersed in boiled water for 15 min. The total protein was separated on 12% gels. The proteins were transferred to polyvinylidene fluoride (PVDF) membranes. Recombinant human MMP-12 was used as positive control. Briefly, after blocking for 1 h at 37°C in 10 mM Tris–HCl pH 7.5, 100 mM NaCl, triethanolamine-buffered saline (TBS) containing 3% bovine serum albumin and 0.1% Tween 20, the membrane was incubated with an antibody (rabbit anti-human MMP-12 mAb, Epitomics) at 1:1000 dilution for 2 h at RT . After repeated washes, the membrane was incubated with Biotin-conjugated goat anti- rabbit IgG (Santa Cruz, American) at 1:1000 dilution for 1 h at RT , followed by incubation with Streptavidin-alkaline phosphatase (R&D, USA) for 1 h at RT. Finally, the membrane was incubated with BCIP/NBT (Calbiochem, Bad Soden, Germany) in the darkness.

### MMP-12 FRET activity assay

The activity of MMP-12 was assessed using a peptide-based FRET assay (EnzoLyte MMP-12 Assay Kit, AnaSpec, Inc, Fremont, CA,USA) in accordance with the manufacturer’s protocols and fluorescence intensity was determined using a fluorescence analyzer (Fluoroskan Ascent 2.6, Thermo) at excitation/emission wavelengths of 340 nm/490 nm. Briefly , aorta wall tissues (200 mg ) in assay buffer(0.5 ml component D, AnaSpec) were ground in liquid nitrogen, centrifuged at 12,000 rpm for 10 min at 4°C and the supernatant collected and stored at −70°C until use. The blood samples(5 ml, no anticoagulant added) were centrifuged at 3000 rpm for 20 min at 4°C and the serum collected and stored at −70°C until use. Samples were incubated with APMA (4-aminophenyl- mercuric acetate, in component C, AnaSpec) at a final concentration of 1 mM in the assay buffer for 2 h at 37°C to activate MMP-12 prior to the experiment. After activating MMPs with APMA for 2 h , blood samples were incubated with 1 μM MMP Inhibitor v (Merck KGaA Frankfurter Str. 250 64293 Darmstadt, Germany), an orally active non-peptidyl hydroxamate compound that acts as an effective broad-spectrum inhibitor against MMP-2, -3,-8,-9, -12, -13, for 1h at 37°C. The assay was carried out in black 96-well microplates. Fluorescence intensities were recorded using a Fluoroskan Ascent fluorimeter at an interval of 5 min over a period of 1 h until peak fluorescence was achieved. The total volume of the assay was 100 μl assay buffer (SensoLyte TM 490 mmp-12 Assay Kit, ANA SPEC) containing various optimized sample volumes.

### Enzyme-linked immuno sorbent assay

Protein samples were prepared by the methods mentioned above. An ELISA kit for MMP-12 detection is commercially available from EIAab (Wuhan EIAab Science Co.,Ltd,China). This assay employs the quantitative sandwich enzyme immunoassay technique. An antibody specific for MMP-12 had been pre-coated onto a microplate. Standards, samples and the antibody that is bound by horseradish peroxidase were pipetted into the wells, which were then incubate and washed. Peroxidase can catalyze the oxidation of (3,3',5,5'-tetramethylbenzidine)TMB into two colored products. The first product is a blue which, if sufficient acid exists, the blue product will be further oxidized to a yellow diimine, that is stable in acidic conditions and has a maximal absorption wavelength of 450 nm. The depth of the color is positively correlated with the concentration of MMP-12. Samples were diluted with the appropriate amounts of sample dilution buffers provided with the kits. All tests were performed in duplicate for each sample. The optical density OD was determined using a microplate reader set to 450 nm, the average OD was calculated for each set of standards.

### Statistical analysis

Analysis was performed by using SPSS version20.0. Descriptive statistics analysis was done. An independent sample *t* test was used to find out the difference between the means in two groups. Values were given as mean±s.e.m. If the difference is P<0.05, then there is statistical significance.

## Results

### Reverse transcription-polymerase chain reaction

**Figure 2:**
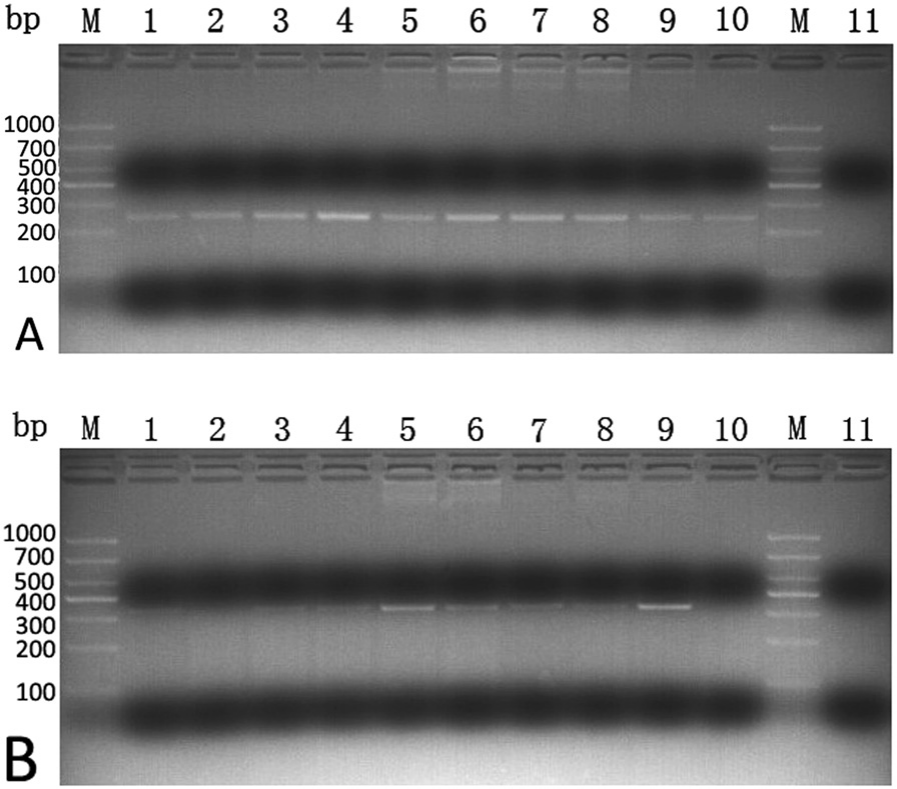
**Agarose gel electrophoresis analysis of PCR fragment obtained from human aorta.** MMP-12 gene fragments were amplified from human aorta wall by RT-PCR. PCR amplifications were then analyzed by electrophoresis using 1% agarose gel stained with Gelview. The gel was viewed on a Tanon 3500R Gel Documentation System and recorded. (**A**) cDNA corresponding to MMP-12 in human aorta was PCR-amplified using primers for MMP-12. (**B**) cDNA corresponding to GAPDH in human aorta was PCR-amplified using primers for GAPDH. Lanes 1–2: Aorta wall of coronary heart disease patient; Lanes 3–10: AD; Lane M: DNA ladder; Lane 11: negative control.

mRNA were isolated from human aorta and then cDNA were synthesized by reverse transcription using the first strand cDNA as templates. The target DNA sequence was amplified by PCR (polymerase chain reaction) with the primers that primed amplification of an 250-base-pair (bp) segment of the MMP-12 gene and a 410-base-pair (bp) segment of the GAPDH gene. The authenticity of amplified PCR products was confirmed by nucleotide sequence analysis. MMP-12 and GAPDH mRNA had been detected by RT-PCR in ascending aorta specimen from AD and CAD patients (Figure 2A~B).

### Immunohistochemical staining

**Figure 3:**
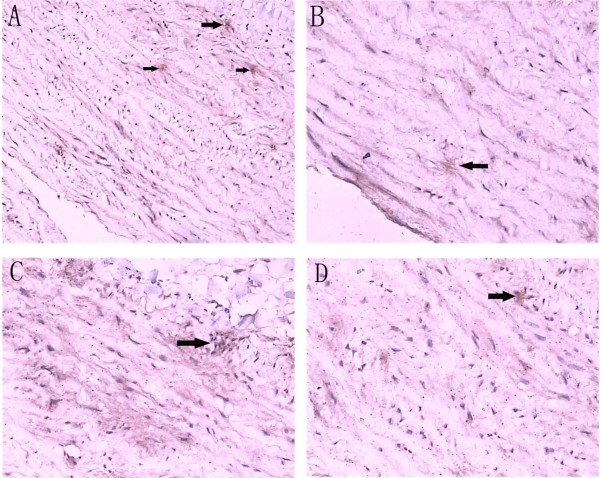
**Immunohistochemical staining.** Ascending aorta specimen in the entry site of the dissection from a AD patients were sectioned and labeled with MMP-12 antibodies as indicated. Sections were developed with alkaline phosphatase anti-alkaline phosphatase (APAAP) techniques and counterstained with hematoxylin. Total cells and positive cells were counted under the microscope. Arrows indicate examples of MMP-12 positive cells, **A** (magnification×200), **B**, **C**, **D** (magnification×400).

The culprit lesion was obtained from a typical patient who underwent aortic replacement. Representative micrograph of MMP-12 immunostained specimens are shown in Figure 3 (A~D). The aorta wall tissues are of loosely structured and disordered. The elastic fibers are broken and disintegrated with inflammatory cells infiltration in the aortic wall. The majority of MMP-12–positive cells were located in deeper parts of the entry site of the dissection.

### Western blotting

**Figure 4:**
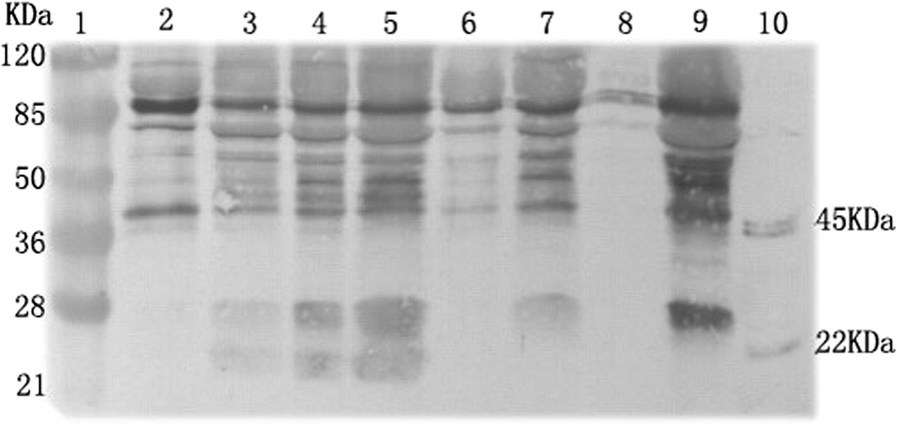
**MMP-12 expression in the human aorta wall.** Lysates from tissues samples collected from AD and non-AD patients were separated on 12% SDS-PAGE gels and were analyzed by immunoblotting using an antihuman MMP-12 monoclonal antibody. Recombinant MMP-12 expressed in *E. coli* was used as positive control. Lane 1 : molecular weight markers; Lane 2–7: Western blot products from the aorta walls of AD patients; Lanes 8–9 : Western blot products from the coronary disease patient’s aorta walls; lane 10: Recombinant human MMP-12.

Recombinant MMP-12 with molecular weights of 45 KDa and 22 KDa could been identified by Western blotting (Figure 4). MMP-12 with molecular weight of 22 kDa could be seen clearly in three cases in the AD group on the basis of calibrated molecular weight markers and recombinant MMP-12, but no expression is found in the CAD group. MMP-12 with molecular weight of 45 kDa is expressed in both groups, yet the expression of MMP-12 with molecular weight of 45 kDa is comparatively lower than that of 22 kDa MMP-12 in the AD group.

### Enzymatic assay of MMP-12 activity using the FRET peptide

**Figure 5:**
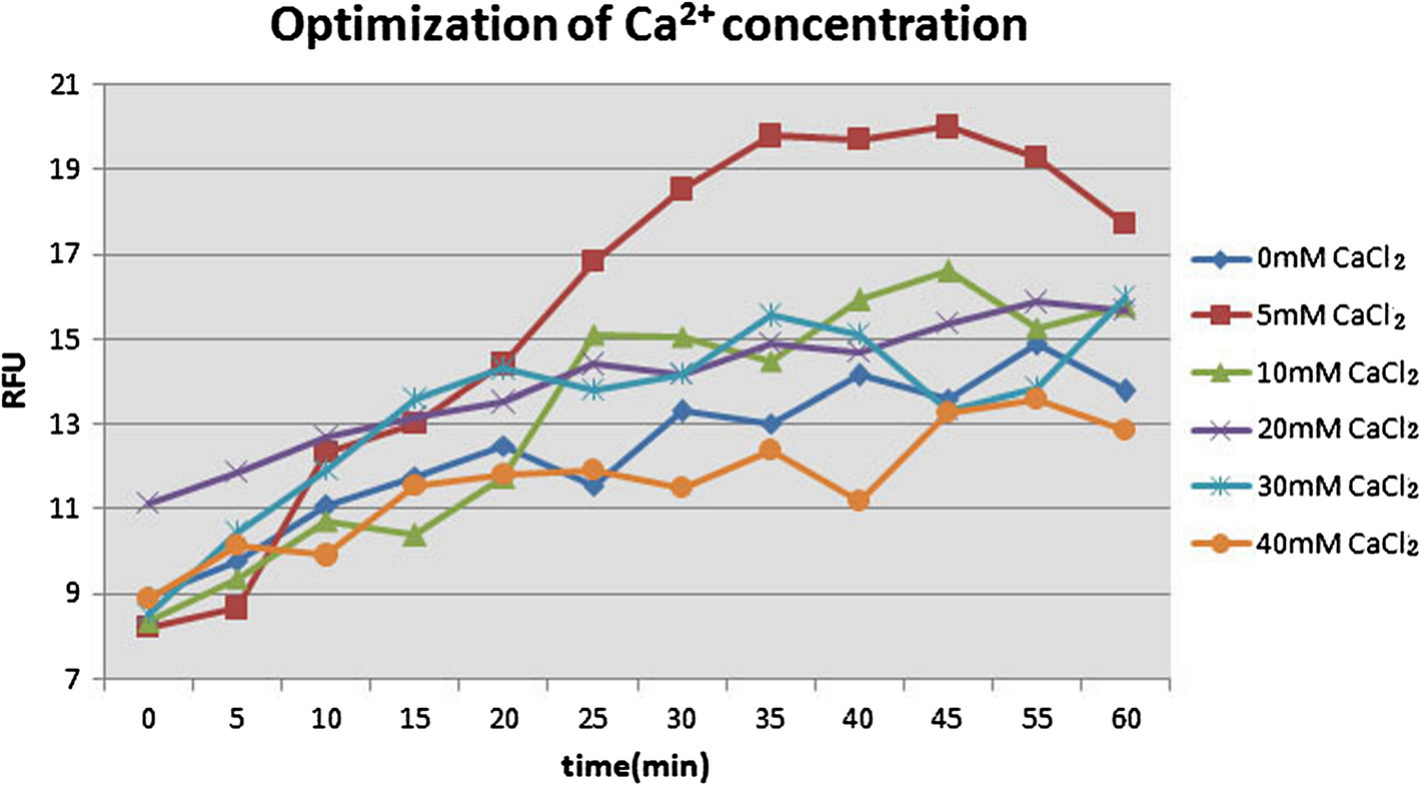
**Effects of different concentration of Ca**^**2+ **^**ions on MMP-12 activity.** The MMP-12 assay was based on the MMP-12 hydrolysis of FRET substrate ( AnaSpec, Inc, Fremont, CA,USA) .A total of 100 μl reaction mixture consisting of 1 μl (100 ng) MMP-12, different concentrations of Ca^2+^ ions (0-40 mM) and 50 μl of buffer ( AnaSpec, Inc, Fremont, CA,USA)were incubated at 37°C for 10 min. Fluorescence was read at Ex/Em wavelengths = 340 nm/490 nm for 60 min. RFU: Relative Fluorescence Unit.

**Figure 6:**
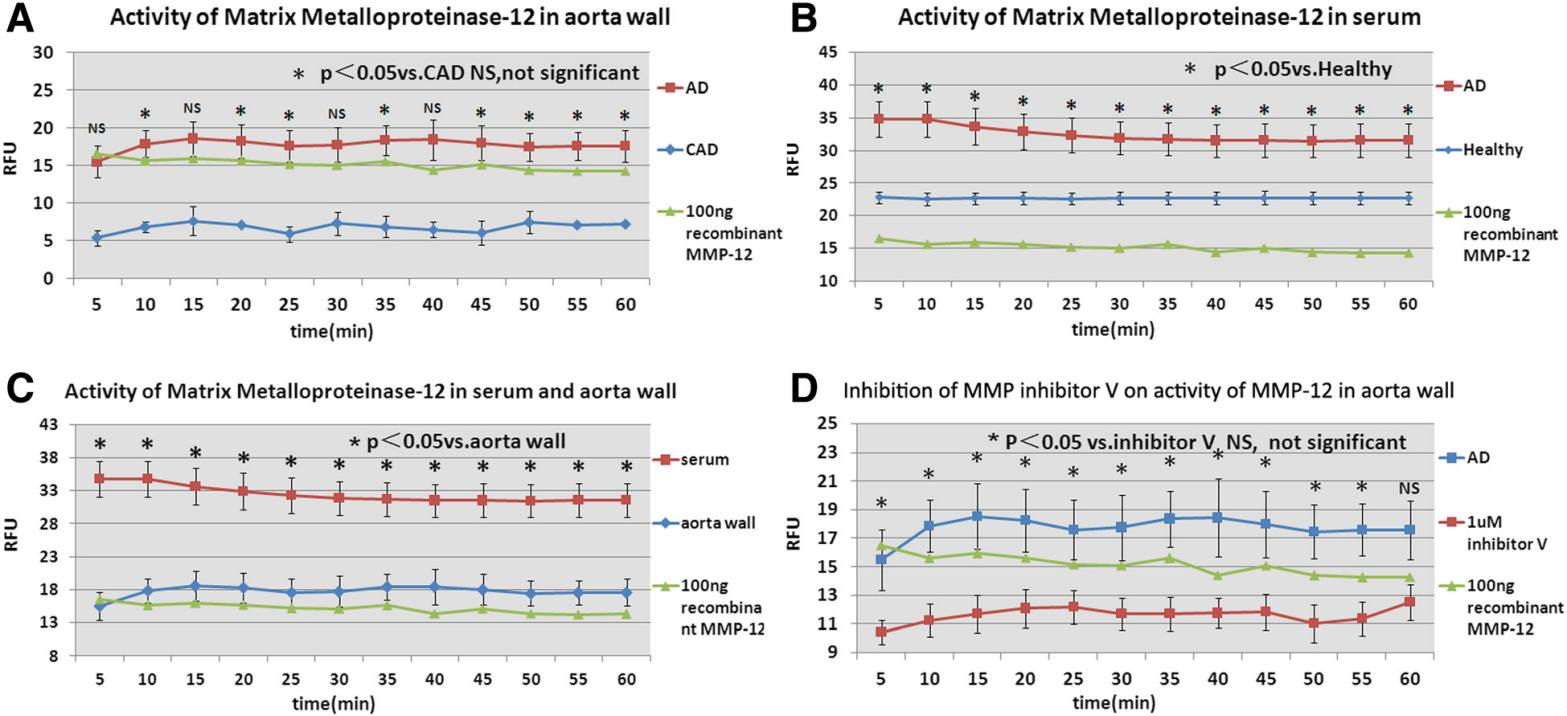
**Continuous assay for MMP-12.** Continuous fluorometric assay of MMP-12 in (**A**) aortic specimen from AD(n=12) and CAD(n=4), (**B**) blood serum sample from AD (n=15) and healthy(n=10) , (**C**) aortic specimens and sera from AD, (**D**) aortic specime n from AD incubated with MMP Inhibitor v. Assays were performed at RT in a total of 100 μl assay buffer containing various optimized sample volumes, by activating MMPs with 1mM APMA , inhibiting MMPs with 1 μM MMP Inhibitor v, or 100 ng of recombinant MMP-12 served as a control. Fluorescence was read at Ex/Em wavelengths = 340 nm/490 nm for 60 min. RFU: Relative Fluorescence Unit; AD: aortic dissection; CAD: coronary artery disease.

Comparing with recombinant MMP-12 which exhibited the highest activity in 5 mM CaCl_2_ (Figure 5), MMP-12 activity levels in induced ascending aorta specimen from 12 AD, 4 CAD, and serum samples from 15 AD and 10 healthy patients were assessed using a FRET assay. Kinetic analysis verified that MMP-12 proteolysis existed in both aortic specimen and serum samples from patients with or without aortic dissection. The result further showed that the concentration of MMP-12 was higher in the aortic wall tissue of the AD group than in CAD group (P <0.05, Figure 6A). Meanwhile, the level of MMP-12 was higher in serum samples of the AD group than in the healthy group (P <0.05, Figure 6B). A comparison of MMP-12 levels in aortic wall tissues and serum samples of the AD group showed that MMP-12 activity was higher in serum samples than in aortic wall tissue (P <0.05, Figure 6C). MMP-12 activity in the aortic wall tissue could be inhibited by MMP inhibitor v (P <0.05, Figure 6D).

### Enzyme-linked immuno sorbent assay result

**Figure 7:**
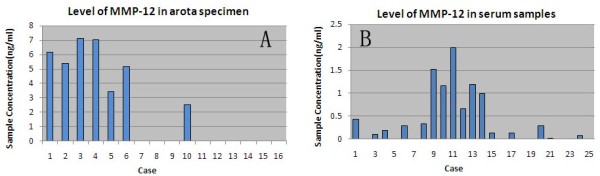
**MMP-12 levels in the aortic specimens and sera.** MMP-12 levels were measured using ELISA as described. (**A**) Level of MMP −12 in the aorta wall. Cases 1–10 are the aorta tissues of AD, and Cases 11–16 are aorta tissues of CAD. (**B**) level of MMP-12 in sera. Cases 1–15 are the sera of AD, and cases 16–25 are sera of healthy patients.

MMP-12 concentrations measured by ELISA were used to assess the levels in ascending aorta specimen from 12 AD, 4 CAD, and serum samples from 15 AD and 10 healthy patients. Higher MMP-12 concentrations were detected in aorta specimen and serum samples of the AD group compared with aorta specimens of the CAD group and serum samples of healthy controls (3.7±0.9, 0.6±0.16 vs. no detectable quantity of MMP-12, 0.05±0.03ng/ml, P<0.05, P<0.05, respectively) (Figure 7).

## Discussion

Acute aortic dissection is the most common catastrophic aortic condition but lacks an effective pharmaceutical therapy. The aortic wall consists of 3 layers: the intima (inner layer made of endothelial cells), media (containing muscular elastic fibers), and adventitia (outer connective tissue). In the human aorta, the elastin-to-collagen ratio decreases in progressively distal regions making the aorta most flexible at proximal regions [24]. Acute dissection is believed to begin with the formation of a tear in the aortic intima, permitting entry of blood to a diseased underlying media characterized by elastic degeneration and smooth muscle cell loss [12,25]. In recent years, the increasing importance of elastine for the development of aortic dissection has come to the fore [12,26]. MMP-12 mRNA was detected in aorta walls by RT-PCR in both the AD and CAD groups. The result demonstrated that the MMP-12 gene is expressed in the human aorta. This observation is consistent with the study by Mohamed and colleagues [27], who compared dissected and normal aortic wall by quantitative reverse transcription-polymerase chain reaction (qRT-PCR). They found a number of matrix metalloproteases (MMP-19, MMP-12, MMP-9) were differentially expressed in acute aortic dissection patients. MMP-19 was upregulated and MMP-12 and MMP-9 were downregulated. Weis-Müller and colleagues [28] also compared tissue from dissected aorta with normal aortic tissue using microarrays andcould not detect MMP-12 expression in the array, but observed a higher gene expression of the matrix metalloproteinases MMP-11, MMP-14 and MMP-19. A possible explanation is that our gene expression studies considered the entry site of the dissection and not the whole aortic wall . we also detected MMP-12 mRNA in ascending aorta specimen obtained from patients operated on for coronary artery bypass surgery. Previous human studies [29,30] have demonstrated that MMP-12 is minimally present in early lesions of atherosclerotic arteries, and strongly expressed in advanced atherosclerotic plaques.

Western blotting confirmed that the 22 kDa MMP-12 was present in the lysate of damaged aorta walls. This is, however, not surprising because MMP-12 is secreted as a pro-enzyme and undergoes self-activation through autolytic processing [31]. Thus, it is likely when aorta walls were damaged, MMP-12 became totally activated and processed into the 22 kDa active form. Recombinant rabbit MMP-12 has been shown to activate other MMPs such as MMP-2 and −3, suggesting that once MMP-12 is activated, there is a cascade of activation of other MMPs that leads to ECM degradation [32].

Clinical and basic studies have implicated changes in MMPs activity in aortic Dissection, but the key effectors and mechanism of action is presently unknown.

Kurihara and colleagues [33] recently reported that MMP-9 was significantly elevated in blood samples from acute aortic dissection patients than in those from the patients with nonruptured chronic aortic aneurysm or healthy volunteers. The acute phase of thoracic aortic dissection is characterized by a prominent increase of MMP-8 plasma levels [34]. Smooth muscle cells in the dissected media increased MMP-2 production, which could promote elastin degradation in thoracic aortic dissection [35]. Low levels of plasma MMP8 can rule out acute aortic dissection in a minority of patients [36]. However, the performance of MMP-12 for the ocurrence of AD is presently unknown.

Accordingly, through the combined use of a fluorogenic substrate and ELISA, the present study demonstrated that a continuous fluorescent signal and the level of MMP-12 could be detected from serum samples of AD. These results indicate that an ambient level of MMP-12 proteolytic activity exists within the blood circulation. With the occurrence of AD, serum MMP-12 activity was transiently increased. Thus, this study demonstrated for the first time that MMP-12 activity exists in the serum of AD. We also identified the presence and activation of MMP-12 within the entry site of aortic dissection, so, the results of the present study set the stage for future mechanistic studies especially in MMP-12 induction and activation on the development of AD.

## Conclusions

These data reported here demonstrated that MMP-12 activity exists in the serum and aorta wall of AD. This new information adds to our understanding of the pathophysiology of the dissection. Measurement of serum MMP-12 levels with a peptide-based FRET assay or ELISA in AD is a simple and relatively rapid laboratory test, that can be used as a biochemical indicator of aortic disease. Further studies are required in order to draw precise conclusions on the clinical significance of serum levels of MMP-12 in diagnosis and prognostic applications in the treatment of this deadly disease.

## Competing interests

The authors declare that they have no competing interests.

## Authors’ contributions

YS and YHX contributed equally to this work. ZHM, RWM , YHX, and YS conceived and designed the study and wrote the manuscript. ZHM and YHX designed the primers and constructed MMP-12 expressive plasmid . RWM , YS, FL and QL collected tissues. RWM, YHX, FL, CZ carried out the immunoassays. CZ, RY, SB, QFY, JXW implemented purifcation and characterization of human MMP-12. FL and HBW performed reverse transcription-polymerase chain reaction. FL, CZ and RY carried out FRET assay . RWM, YHX, QL and YS participated in the experimental data collection and analysis. YS, YHX and FL performed the statistical analysis. All authors read and approved the final manuscript.

## Pre-publication history

The pre-publication history for this paper can be accessed here:

http://www.biomedcentral.com/1471-2261/13/34/prepub
